# Combined subtotal gastrectomy and splenectomy after partial splenic embolization for a patient with gastric cancer and immune thrombocytopenic purpura: A case report

**DOI:** 10.1016/j.ijscr.2019.08.027

**Published:** 2019-08-31

**Authors:** Yuki Kaneko, Shin Saito, Daijiro Takahashi, Takashi Ui, Hidenori Haruta, Kentaro Kurashina, Hironori Yamaguchi, Yoshinori Hosoya, Joji Kitayama, Alan Kawarai Lefor, Naohiro Sata

**Affiliations:** Department of Surgery, Jichi Medical University, Tochigi, Japan

**Keywords:** ITP, immune thrombocytopenic purpura, *H. pylori*, *Helicobacter pylori*, Immune thrombocytopenic purpura, Gastric cancer, Partial splenic embolization, Case report

## Abstract

**Introduction:**

Immune thrombocytopenic purpura is an acquired thrombocytopenia. Preoperative management of thrombocytopenia is important in patients with gastric cancer. Partial splenic embolization can be effective for patients with thrombocytopenia, but could lead to ischemic necrosis of the remnant stomach when performing subtotal gastrectomy with splenectomy.

**Presentation of case:**

The patient is an 84-year old woman evaluated for anemia. Endoscopy revealed an advanced gastric cancer with bleeding. The patient also had immune thrombocytopenic purpura with a platelet count <50,000/μL. Administration of platelets did not increase the platelet count. Partial splenic embolization was performed followed by administration of high-dose immunoglobulin. The platelet count was over 50,000/μL preoperatively. The patient underwent combined subtotal gastrectomy and splenectomy, followed by an uneventful course.

**Discussion:**

Patients with immune thrombocytopenic purpura and advanced gastric cancer can have anemia. Partial splenic embolization has been used to treat patients with refractory immune thrombocytopenic purpura as an alternative to splenectomy. Preoperative partial splenic embolization and high-dose immunoglobulin therapy resulted an increased platelet count in this patient. Elderly patients with gastric cancer have a high risk of postoperative complications. Patients with gastric cancer undergoing total gastrectomy have an impaired postoperative quality of life compared to those who undergo subtotal gastrectomy. We performed a subtotal gastrectomy and splenectomy as a function-preserving operation, completed safely by maintaining blood flow to the remnant stomach.

**Conclusion:**

Partial splenic embolization is effective for patients with immune thrombocytopenic purpura and gastric cancer. Combined subtotal gastrectomy and splenectomy is achieved by preserving blood flow to the remnant stomach.

## Highlights

• Immune thrombocytopenic purpura (ITP) is an autoimmune disease characterized by thrombocytopenia.
• Gastric cancer complicated by ITP can result in gastrointestinal bleeding.
• Partial splenic embolization effectively treats thrombocytopenia.
• Preserving blood flow to the remnant stomach is important when performing combined subtotal gastrectomy and splenectomy.

## 1 Introduction

Immune thrombocytopenic purpura (ITP) is an acquired thrombocytopenia caused by immune destruction of platelets in the spleen and results in variable bleeding symptoms [1]. Preoperative management of thrombocytopenia and anemia can be crucial for patients with gastric cancer. Partial splenic embolization has been shown to be effective to increase the platelet count [2]. Splenectomy is performed in patients with ITP who do not respond to medical treatment, which usually includes corticosteroids [3]. However, ischemic necrosis of the remnant stomach could be a serious complication of subtotal gastrectomy with splenectomy [4].

We report an elderly patient with gastric cancer complicated by ITP who underwent subtotal gastrectomy and splenectomy after partial splenic embolization. Combined subtotal gastrectomy and splenectomy was performed safely by meticulously preserving blood flow to the remnant stomach. The work has been reported in line with the SCARE criteria [5].

## 2 Presentation of case

**Fig. 1:**
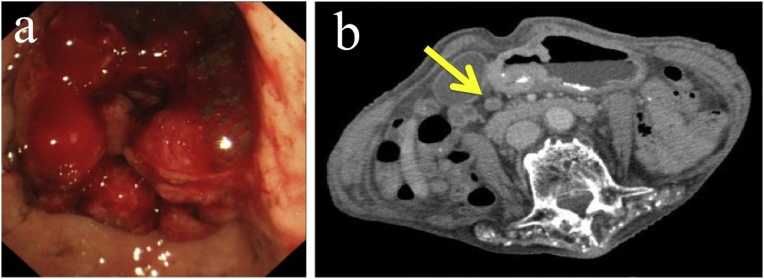
(a) Upper gastrointestinal endoscopy showed a Borrmann type3 tumor with pyloric stenosis and bleeding. (b) Contrast-enhanced computed tomography scan showed lymph node involvement around the stomach but no obvious distant metastases.

An 84-year-old Japanese woman was referred with a history of progressive anemia. Endoscopic examination showed a Borrmann Type 3 tumor with pyloric stenosis, which result in bleeding (Fig. 1a). Biopsy of the tumor revealed adenocarcinoma. Abdominal contrast-enhanced computed tomography scan showed a tumor in the pyloric region and enlarged pyloric lymph nodes consistent with metastases (Fig. 1b). There was no evidence of distant metastatic disease.

**Fig. 2:**
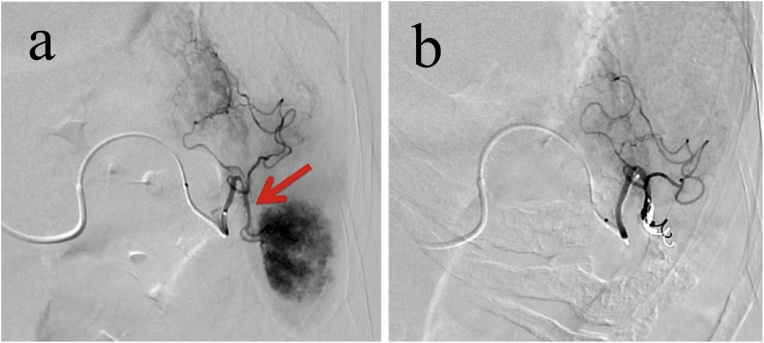
Images from the partial splenic embolization performed by trans-catheter embolization of the inferior branch of the splenic artery. (a) Pre-embolization. (b) Post embolization.

**Fig. 3:**
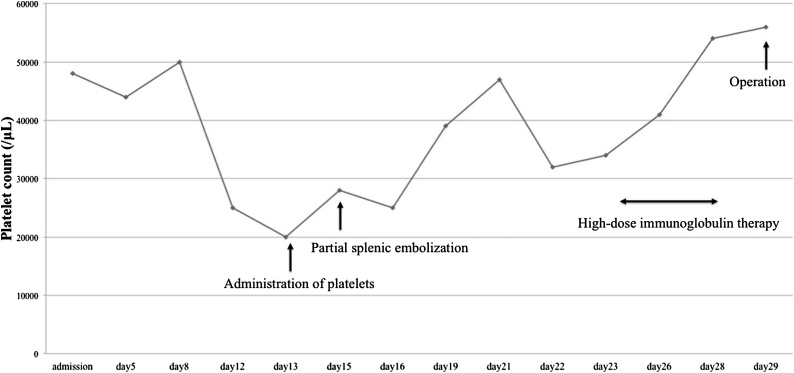
Change in platelet count over time. The platelet count increased to 56,000/μL on the day of the gastric resection after partial splenic embolization and high-dose immunoglobulin therapy.

The patient was also diagnosed with ITP and the platelet count was <50,000/μL. Former *Helicobacter pylori* (*H. pylori*) eradication and oral corticosteroid therapy failed to improve her platelet count. Thrombocytopenia and anemia deteriorated and resulted in tachycardia. Administration of platelets was performed in an attempt to increase the platelet count but was ineffective. Partial splenic embolization was performed 14 days before the planned gastric resection followed by immunoglobulin therapy. This was performed by trans-catheter embolization of the inferior branch of the splenic artery (Fig. 2). The platelet count was >50,000/μL at the time of gastric resection (Fig. 3).

The patient underwent a subtotal gastrectomy with a Roux-en-Y reconstruction and simultaneous splenectomy. We preserved the ascending branch of the left gastric artery, the short gastric artery, the posterior gastric artery, and the left gastroepiploic artery during subtotal gastrectomy to maintain blood flow to the remnant stomach. Histopathological examination showed a well- to moderately- differentiated gastric adenocarcinoma which had reached the serosal layer with lymph node metastases. According to the TNM classification, the tumor was stage IIIC (T4a, N3a (8/17), M0).

The patient had an uneventful course and was discharged on the 18th postoperative day. Postoperative adjuvant chemotherapy was not given due to her advanced age. There was no recurrence at 32 months after surgery and the platelet count returned to normal levels. To the best of our knowledge, this is the first report of a patient with gastric cancer who underwent combined subtotal gastrectomy and splenectomy after preoperative partial splenic embolization for ITP.

## 3 Discussion

ITP is an acquired thrombocytopenia caused by immune destruction of platelets and results in variable bleeding symptoms [1]. Bleeding is a major cause of morbidity and mortality in patients with ITP [6]. The severity of illness and overall condition of the patient must be considered when evaluating treatment options for patients with ITP, because the risk of bleeding increases with age [7]. Corticosteroids are the most common first line therapy for patients with ITP, which is sometimes accompanied by simultaneous intravenous immunoglobulin therapy. Splenectomy is a second line approach in patients refractory to medical management [8]. The eradication of *H. pylori* is recommended for *H. pylori*-associated ITP [9]. The present patient had an advanced gastric cancer and ITP, which caused bleeding and pyloric stenosis and we considered strategies for both advanced gastric cancer and ITP.

Preoperative management of thrombocytopenia is important for patients with ITP and gastric cancer. High-dose immunoglobulin therapy, administration of platelets, and partial splenic embolization have been reported to increase the platelet count before surgery [10,11]. Platelet transfusion for the management of ITP remains controversial and is recommended only in patients with catastrophic hemorrhage or who undergo invasive surgical procedures [12]. Although it has been suggested that laparoscopic splenectomy can be safely performed even with thrombocytopenia in patients with ITP, there is no data regarding gastrectomy in these patients and therefore we sought to increase the platelet count to reduce the risk of complications from bleeding [13].

Partial splenic embolization was developed to treat hypersplenism and portal hypertension. This procedure has been recently applied to the treatment of patients with refractory ITP as an alternative to splenectomy. Togasaki et al., reported that the median time to achieve the peak platelet count was 13 days after partial splenic embolization in their study on the efficacy of partial splenic embolization for patients with ITP [14]. Partial splenic embolization 14 days prior to surgery and high-dose immunoglobulin therapy just before operation resulted in increasing the platelet count >50,000/μL.

Surgical strategies are important for patients with gastric cancer and ITP, especially in the elderly. Takeuchi et al., reported that total gastrectomy is significantly associated with severe complication in the elderly [15]. Patients with gastric cancer who underwent total gastrectomy have a significantly impaired quality of life postoperatively compared to patients who underwent subtotal gastrectomy [16]. However, ischemic necrosis of the remnant stomach would be a serious complication after performing subtotal gastrectomy with splenectomy [4]. Total gastrectomy is usually recommended due to the risk of such a serious complication. When performing combined subtotal gastrectomy and splenectomy, assuring blood flow to the remnant stomach is essential. Subtotal gastrectomy with radical lymph node dissection and splenectomy was performed as function-preserving surgery in this patient, and was performed safely by preserving the ascending branch of the left gastric artery, the short gastric artery, the posterior gastric artery, and the left gastroepiploic artery.

Preoperative management and surgical strategies must be selected according to the condition of the individual patient. Partial splenic embolization can be effective as preoperative therapy for thrombocytopenia in patients with ITP who do not respond to corticosteroids. Preserving the blood flow to remnant stomach is important when performing combined subtotal gastrectomy and splenectomy.

## 4 Conclusion

ITP is an acquired thrombocytopenia caused by immune destruction of platelets in the spleen. Gastric cancer complicated by ITP can result in gastrointestinal bleeding. We report an elderly patient with a gastric cancer complicated by ITP treated with combined subtotal gastrectomy and splenectomy after partial splenic embolization. Partial splenic embolization was effective for the treatment of thrombocytopenia. Ischemic necrosis of the remnant stomach would be a serious complication after performing subtotal gastrectomy with splenectomy. We meticulously preserved blood flow to remnant stomach with a view to achieving this combined operation without impairing the efficacy of lymph node dissection.

## Funding

All authors have no funding regarding this paper.

## Ethical approval

The need for ethical approval for this paper was waived by the committee of Jichi Medical University Hospital.

## Consent

Written informed consent was obtained from the patient for publication of this case report and accompanying images.

## Author’s contribution

All authors in this manuscript contributed to the interpretation of data, and drafting and writing of this manuscript. YK, SS, DT, TU, HH, KK, HY and YH were engaged in patient's care in her hospital coarse including surgery and endoscopy under the supervision of JK, AL and NS. AL helped in drafting the manuscript and interpretation of data. All authors have read and approved this manuscript for publication.

## Registration of research studies

The name of registry is research registry, and the unique identifying number (UIN) we obtained is researchregistry4925.

## Guarantor

Dr. Sata, who is the president of Jichi Medical University Hospital, is the Guarantor.

## Provenance and peer review

Not commissioned, externally peer-reviewed.

## Declaration of Competing Interest

All authors declare no conflicts of interests regarding the publication of this paper.
